# Corrosion Behavior of Cobalt Oxide and Lithium Carbonate on Mullite–Cordierite Saggar Used for Lithium Battery Cathode Material Sintering

**DOI:** 10.3390/ma16020653

**Published:** 2023-01-09

**Authors:** Zhenhua Sun, Shaopeng Li, Huiquan Li, Mingkun Liu, Zhanbing Li, Xianjie Liu, Mingyong Liu, Qiyun Liu, Zhaohui Huang

**Affiliations:** 1 School of Materials Science and Technology, Beijing Key Laboratory of Materials Utilization of Nonmetallic Minerals and Solid Wastes, National Laboratory of Mineral Materials, China University of Geosciences (Beijing), Beijing 100083, China; 2 CAS Key Laboratory of Green Process and Engineering, National Engineering Research Center of Green Recycling for Strategic Metal Resources, Institute of Process Engineering, Chinese Academy of Sciences, Beijing 100190, China; 3 School of Chemical Engineering, University of Chinese Academy of Sciences, Beijing 100149, China

**Keywords:** lithium battery cathode material, saggar, corrosion behavior, penetration, volume expansion

## Abstract

Mullite–cordierite ceramic saggar is a necessary consumable material used in the synthesis process of LiCoO_2_ that is easily eroded during application. In our study, we systematically investigated the characteristics and surface corrosion behavior of waste saggar samples. We divided the cross sections of waste saggar into the attached layer, hardened layer, permeability layer, and matrix layer. Then, we examined the high-temperature solid-state reactions between saggar powder and lithium carbonate or cobalt oxide to identify erosion reactants correlating with an increase in the number of recycled saggars. The results of time-of-flight secondary ion mass spectrometric analysis (TOF-SIMS) prove that the maximum erosion penetration of lithium can reach 2 mm. However, our morphology and elemental distribution analysis results show that the erosion penetration of cobalt was only 200 μm. When enough lithium carbonate reacted, lithium aluminate and lithium silicate were the main phases. Our X-ray computed tomography (X-ray CT) analysis results show that the change in phase volume before and after the reaction, including the generation of oxygen and carbon dioxide gas, led to the internal crack expansion of the material–saggar interface. Our results can contribute to improving saggar and upgrading waste saggar utilization technology.

## 1. Introduction

Lithium-ion batteries (LIBs) have been broadly used in new energy vehicles and 3C products (computers, communication devices, and consumer electronics), and their estimated output value is expected to approach USD 139.36 billion by 2026 [1,2,3]. Lithium cobalt oxide (LiCoO_2_) is a prime battery cathode material for 3C products due to its high specific energy (145 mAhg^−1^) and excellent cycle life since J. B. Goodenough first identified and developed Li_x_CoO_2_ as a cathode material [4,5]. LiCoO_2_ cathode materials are mainly produced by solid-state sintering reactions in a roller kiln at approximately 1000–1200 °C; the raw reaction materials are packed in ceramic saggars [6]. Ceramic saggars are classified as consumables because they are eroded by the calcining process of cathode materials, and their recycled service-life is approximately 10–30 cycles [7]. Mullite–cordierite saggars are mainly used in the production of LiCoO_2_ cathode materials because of their low cost, considerable high-temperature mechanical properties, and their low thermal expansion coefficient [7,8]. With the increase in their usage duration, reactions between LiCoO_2_ cathode materials and mullite–cordierite saggars result in refractory corrosion and peeling, consequently contaminating the cathode materials [9]. In addition, waste saggars increase the raw-material reaction loss of the cathode, which increases production and waste treatment costs. During actual production, approximately 20–30 saggars are consumed for 1 ton of LiCoO_2_ cathode materials. With the continuous increase in the demand for LiCoO_2_ cathode material, the demand, output, and consumption of saggars, as well as the production of waste saggars, will continue to increase. Research on corrosion behavior has contributed to the subsequent development of new corrosion-resistant saggars, in addition to the utilization of waste saggars.

LiCoO_2_ product quality is considerably affected by saggar quality. Therefore, it is necessary to study the erosion mechanism of mullite–cordierite saggar during LiCoO_2_ synthesis. Zhai et al. studied saggar corrosion in the preparation of lithium battery cathodes in mullite-based saggar refractories during the production of Li(Ni_x_Co_y_Mn_z_)O_2_(LNCM) material with increasing calcining temperature and found that LiAlSiO_4_, LiAlSi_2_O_6_, LiAlO_2_, and NiAl_2_O_4_ spinel-based solid solutions are formed from the interactions between LNCM materials and mullite-based saggars [8]. Huang et al. suggested that the Li-containing compound first reacts with the magnesia alumina spinel ceramic and produces a Li_x_M_y_O_z_ (M = Al/Mg) compound as the byproduct; the Li_x_M’_y_O_z_ (M’ = Al/Mg/Co) compound is then generated as a result of the further permeation of a Co-containing compound, which is separated from the Li_x_M_y_O_z_ (M =Al/Mg) compound [10]. Duan et al. conducted corrosion tests of cordierite–mullite and SiC refractory slabs at 780 °C for 20 h and found that a large number of reactants were produced, leading to the failure of cordierite–mullite refractories [7]. MgO-based refractories and KAlSi_2_O_6_-containing materials have been used to improve corrosion resistance to LIB materials, extending the service life of refractories [9,11,12]. The reactions between the raw materials of lithium compounds and ceramics are the main reason for discarding mullite ceramic saggars. However, the synthesis temperature of LiCoO_2_ is higher than that of LNCM, and the raw materials are different, meaning that the above research results inadequately explain the corrosion behavior of mullite–cordierite ceramic saggars during LiCoO_2_ preparation. In addition, it is difficult to visually observe lithium using conventional characterization equipment. Hence, many erosion studies have been conducted according to speculation based on indirect evidence. As such, new detection and experimental methods are needed to study the erosion process, examining the increasing recycling times and corrosion behaviors of lithium and cobalt in saggars.

In this work, we collected samples of new and waste saggars to study the phase-change and final corrosion behavior of lithium. We used X-ray CT analysis to non-destructively detect changes in the internal density of saggars before and after use. In addition, we used time-of-flight secondary ion mass spectrometric (TOF-SIMS) analysis to determine the internal differences and changes in lithium content. We also designed high-temperature reaction experiments using different amounts of new saggar powder with lithium carbonate and cobalt tetroxide to verify the erosion reaction behavior of lithium and cobalt on ceramic saggar under different conditions. Next, we investigated the erosion process of saggars during the preparation of cathode materials by performing a thermodynamic analysis of different minerals. We obtained further verification by calculating high-temperature solid-state thermodynamics. Waste saggars are potentially hazardous waste due to their alkaline nature and composition of cobalt oxides and fine particles. Our study on waste-saggar phase composition and erosion mechanisms will aid in developing new corrosion-resistant saggars, as well as realizing the resource utilization of waste saggars.

## 2. Materials and Methods

### 2.1. Material Preparation

We collected new mullite–cordierite ceramic saggars from a saggar manufacturer in Changsha, Hunan Province, China. We collected waste saggars from a LiCoO_2_ cathode manufacturer in Changsha, Hunan Province, China. We purchased lithium carbonate (Li_2_CO_3_, ≥98%, AR) and cobalt tetroxide (Co_3_O_4_, ≥98%, AR) from Xilong Chemical Co., Ltd., Guangdong Province, Guangzhou, China.

First, we crushed the new saggars to 200 mesh powder, mixed the powder with lithium carbonate and cobalt tetroxide, and calcinated the mixture in a muffle furnace at 1000 °C for 8 h to investigate the corrosion of the mullite–cordierite ceramic saggar during the production of LiCoO_2_ cathode materials. The experimental scheme is listed in the following Table 1.

### 2.2. Sample Characterization Techniques

After crushing and grinding, we analyzed the powder scraped from the waste saggar and the powder obtained after the reaction using an X-ray diffractometer (XRD, Empyrean, PANalytical, Almelo, Netherlands. Cu Kα, 40 kV, 40 mA) in a 2θ range from 5° to 90°. We used X’Pert HighScore Plus 2.0 software to determine the phase type and the contents of different components. We cut the new and waste saggar into 10 mm × 10 mm × 18 mm (length × width × height) samples. We gold-coated the polished refractory specimens for microstructural and compositional analyses by scanning electron microscope (SEM, FEI MLA Quant 250, Hillsboro, OR, USA) in BSE mode at an acceleration of 25 kV. We analyzed the distribution of silicon, aluminum, magnesium, and cobalt in different regions on the sample by energy-dispersive X-ray spectroscopy (EDS, EDAX). Then, we analyzed the distribution of lithium by time-of-flight secondary ion mass spectrometry (TOF-SIMS 5-100, ION-TOF GmbH, Münster, Germany). The density distribution and variation of the new saggar and the waste saggar samples were analyzed by X-ray computed tomography (X-ray CT, Xradia 410 Versa, Carl Zeiss, Oberkochen, Germany) to study the permeability of the lithium. Finally, we completed a thermodynamic calculation of the solid-phase reaction using HSC 6.0 Chemistry software.

## 3. Results

### 3.1. Phase Analyses of New and Waste Saggar

**Figure 1:**
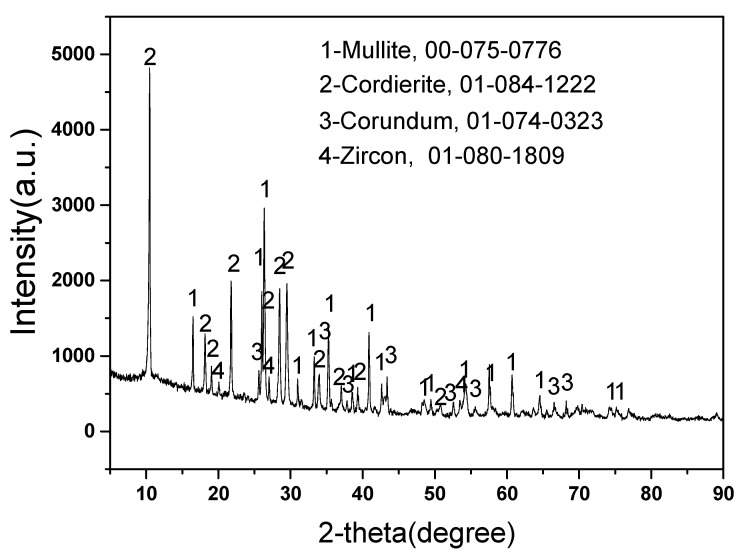
XRD pattern of new mullite–cordierite ceramic saggar (with relative ICDD PDF-4 card numbers).

Figure 1 displays the phases content of new mullite–cordierite ceramic saggar. The new mullite–cordierite ceramic saggar is white and box-shaped. Mullite, cordierite (magnesium aluminosilicate), corundum, and zircon are the main phases of raw saggar, and their contents are 51%, 34%, 14%, and 1%, respectively. Mullite has a low thermal expansion coefficient and high mechanical strength, whereas cordierite also has a low thermal expansion coefficient; therefore, mullite–cordierite ceramic has a low thermal expansion coefficient and excellent thermal shock resistance, which are suitable for the high-temperature solid-state synthesis of cathode materials for lithium batteries [7,13]. A small amount of zircon is added to mullite–cordierite ceramics to increase their strength and creep-resistance temperature [14,15].

**Figure 2:**
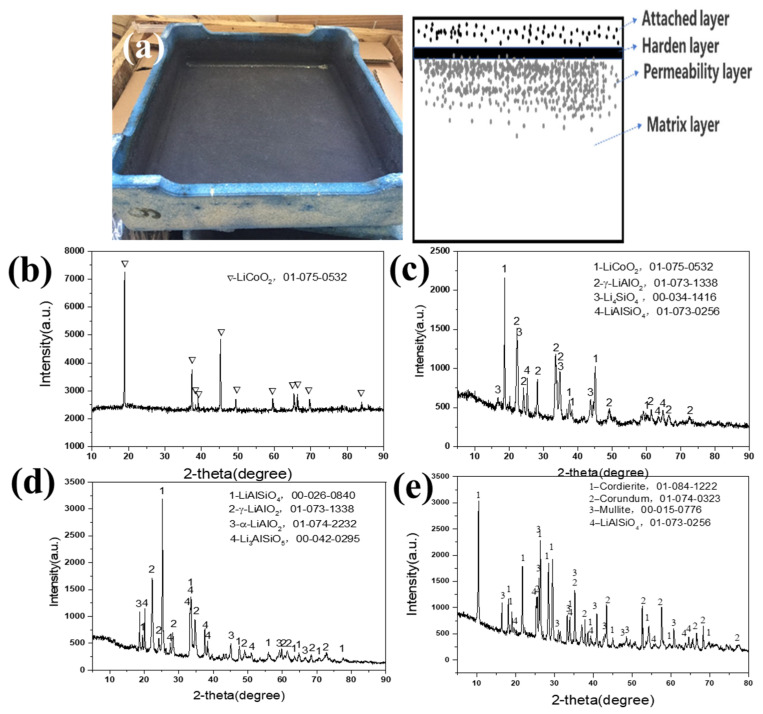
XRD patterns of different regions of waste saggar produced during the synthesis of LiCoO_2_ cathode material. (**a**) Photo of real waste saggar and a section diagrammatic drawing; (**b**) XRD pattern of the attached layer; (**c**) XRD pattern of the hardened layer; (**d**) XRD pattern of the permeability layer; (**e**) XRD pattern of the matrix layer.

Figure 2 shows the waste mullite–cordierite ceramic saggar phases after the LiCoO_2_ cathode material synthesis process. The new mullite–cordierite ceramic saggar becomes waste due to cracking and internal surface slag loss after 10–20 cycles. After sintering, the mullite–cordierite ceramic saggar undergoes a corrosion reaction, and the inner surface of the waste saggar turns black, mainly due to the residual LiCoO_2_ cathode powder; the outer surface was light blue, which may be due to the reaction between saggar and cobalt oxide. After physical separation, the whole saggar interface is divided into four layers, namely an attached layer, hardened layer, permeability layer, and matrix layer. Our XRD analysis shown in Figure 2 demonstrates that the attached layer primarily comprises the uncleaned positive LiCoO_2_ cathode materials; the hardened layer mainly comprises LiCoO_2_ cathode materials γ-LiAlO_2_, Li_4_SiO_4_, and LiAlSiO_4_; and the permeability layer is mainly composed of LiAlSiO_4_, γ-LiAlO_2_, α-LiAlO_2_, and a small amount of Li_3_AlSiO_5_. Most matrix-layer phases are consistent with those of the new saggar, except for the small amount of LiAlSiO_4_ formed by the high-temperature solid-state reaction.

### 3.2. Analysis of Micromorphology and Elemental Distribution

**Figure 3:**
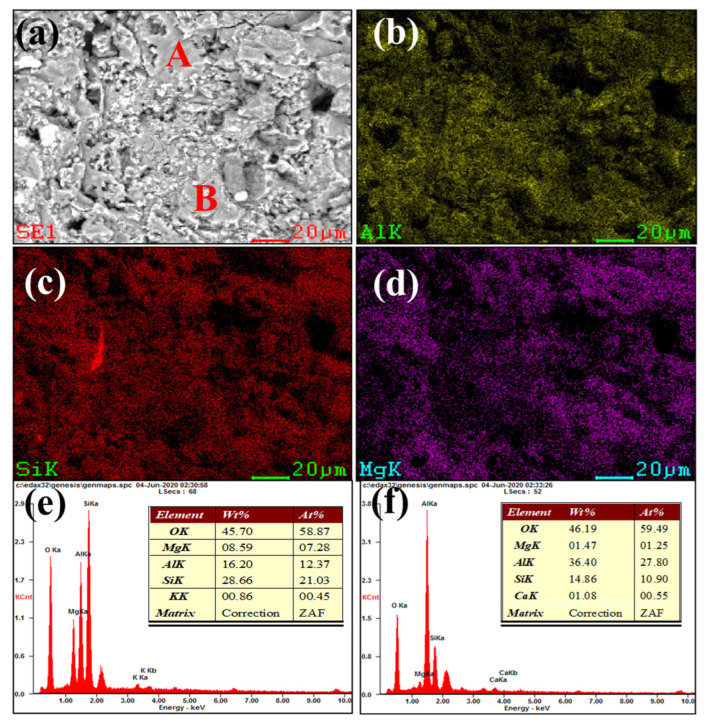
SEM and EDS mapping photos of a section of new saggar. (**a**) SEM image of a sample magnified 1600 times; (**b**), (**c**), and (**d**) EDS mappings of Al, Si, and Mg elements, respectively, in (**a**). (**e**) and (**f**) EDS images of positions A and B, respectively (**a**).

**Figure 4:**
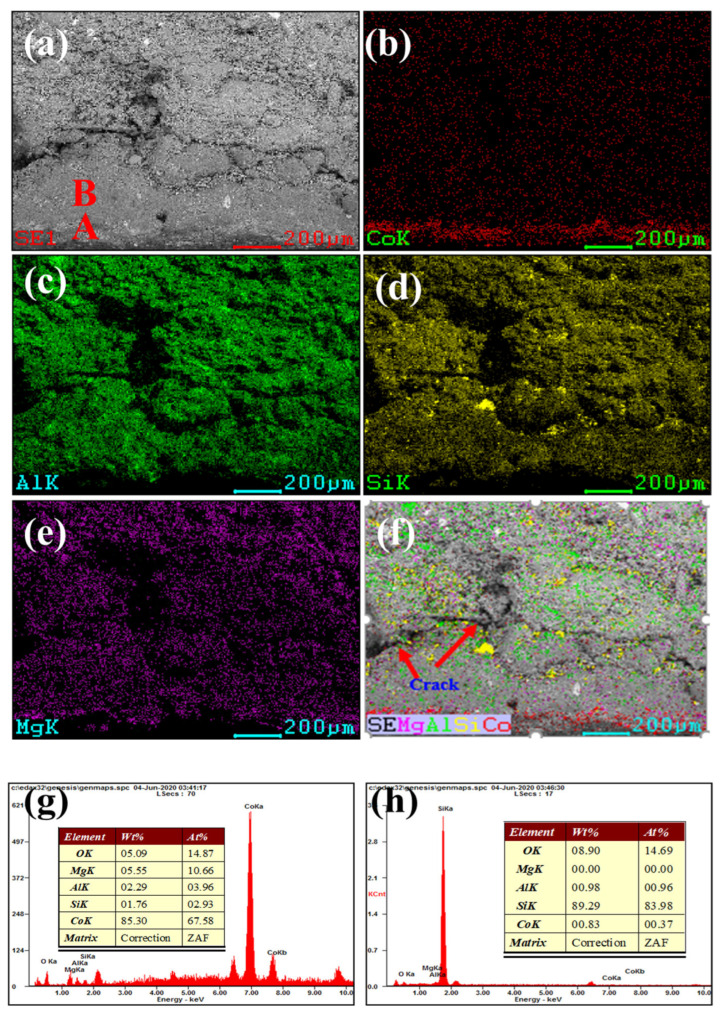
SEM and EDS mapping photos of a section of waste saggar. (**a**) SEM image of a sample magnified 200 times; (**b**), (**c**), (**d**), and (**e**) EDS mappings of Co, Al, Si, and Mg elements, respectively, in (**a**); (**f**) composite of (**a**–**e**); (**g**) and (**h**) EDS images of positions A and B, respectively, in (**a**).

Figure 3 and Figure 4 show our microstructural and elemental distribution analysis of the internal and corrosion interface of new saggar and waste saggar, respectively. We found that the structure was relatively uniform, with massive aggregate closely combined with the reaction material. A small amount of silicon was enriched, and most of the silicon was positively correlated with aluminum and magnesium. Our results show that aluminum and silicon are enriched in some regions, and aluminum, silicon, and magnesium are basically uniformly distributed. For example, position A in Figure 3a shows the enrichment of aluminum, silicon, and magnesium elements, which may be a cordierite phase according to our XRD analysis results; position B is mainly enriched with aluminum and silicon elements (Figure 3f), which may be a mullite phase according to our XRD analysis results. In addition, a small amount of silicon and aluminum show separate enrichment, possibly related to a small amount of quartz and corundum phases. Figure 4 shows that reaction layers can be found on the ceramic surface after the high-temperature sintering reaction, with cracks generated between the body and the reaction layers, possibly because of the phase volume expansion before and after the reaction. According to the elemental distribution of cobalt, aluminum, silicon, and magnesium, cobalt is mainly concentrated on the surface of the attachment layer and is the product of the reaction of Li_2_CO_3_ and Co_3_O_4_ with ceramics during the preparation of the LiCoO_2_ cathode material. As shown in Figure 4b,g, we observed very high cobalt content, indicating that the erosion of cobalt mainly occurs in the surface area, with cobalt struggling to penetrate into the ceramic body. According to Figure 4h, position B primarily consists of silicon, with low cobalt, aluminum and magnesium contents, which may be a generated lithium silicate phase according to our XRD analysis results. Our SEM-EDS analysis results show that the depth of the corrosion layers ranges from 200 to 500 μm. Our results also show that the oxide permeability of cobalt is poor, whereas the changes in the internal silicon and aluminum indicate that an internal high-temperature reaction also occurred.

**Figure 5:**
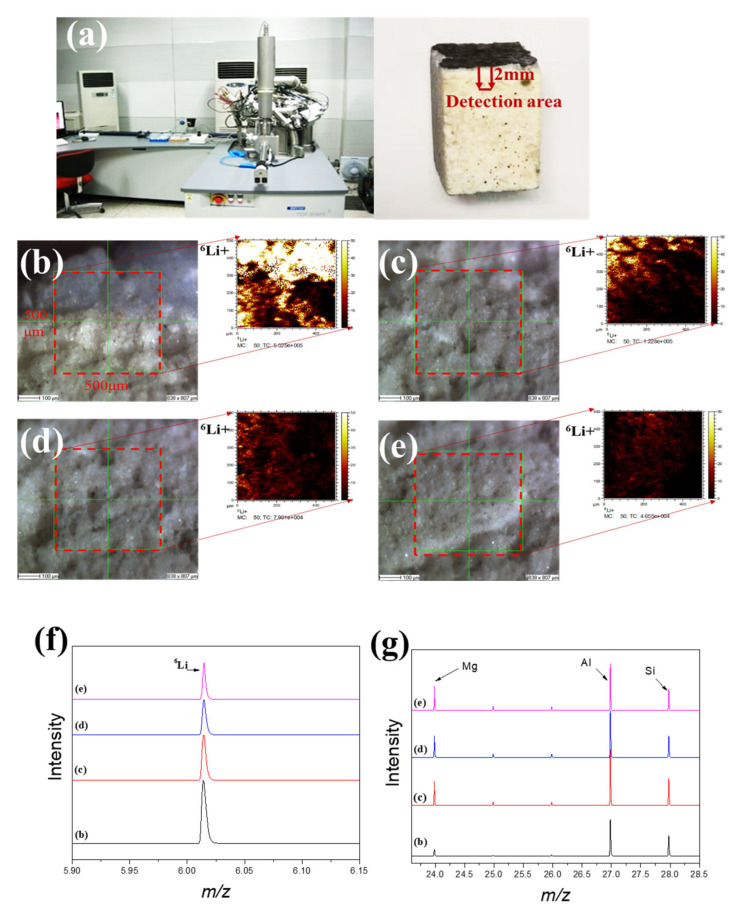
TOF-SIMS lithium permeation analysis of waste saggar. (**a**) TOF-SIMS instrument and analysis sample; (**b**–**e**) local scanning images and lithium intensity distribution maps, respectively, obtained from the surface of the adhesive layer of waste saggar in a scanning analysis area of 500 μm × 500 μm. (**f**) and (**g**) Relative content changes in Li, Al, Si, and Mg in four different regions of b, c, d, and e, respectively.

Figure 5a shows that time-of-flight secondary ion mass spectrometry (TOF-SIMS 5-100, ION-TOF GmbH) is one of the most highly sensitive surface analytical tools that can analyze almost all elements and their isotopes, including hydrogen and lithium, with a detection limit of ppm or ppb; this method can also determine the concentration distribution of trace elements and compounds from the surface to a depth of tens of microns [16,17,18]. Figure 5b–e show the ^6^Li detection intensity distribution of waste saggar from the corroded surface to the interior, respectively. The results show that lithium can be detected at different internal depths, indicating its strong corrosion permeability. In addition, because lithium carbonate is one of the raw materials involved in the high-temperature synthesis of lithium cobalt oxide, its melting point is 723 °C. Therefore, lithium carbonate easily becomes liquid at high temperatures (about 1000 °C) and penetrates the body of the saggar to further react with the ceramic. When the internal penetration of the waste saggar gradually deepened from the corrosion layer to the inside, the strength value of lithium element gradually decreased. With an increase in the number of saggar cycles at high temperatures, the lithium penetration reaction gradually increases and further involves the ceramic body to form a lithium-containing phase. The maximum penetration depth of lithium in ceramic can reach 2 mm. Figure 5f shows that the distribution of ^6^Li gradually decreases from the surface to the interior. In addition to the surface, the changes in lithium, silicon, and aluminum contents represent a positive correlation trend related to the existence of LiAlO_2_, LiAlSiO_4_, and Li_4_SiO_4_ in the XRD analysis results (Figure 5g). Figure 5g also shows that the content of magnesium in the surface is low, with the content of magnesium in the interior unchanged. Our findings indicate that lithium and magnesium may not participate in the reaction and that the main products generated in the surface were the LiCoO_2_ cathode material or lithium reacting with aluminum and silicon. These results are consistent with those obtained by XRD, SEM-EDS, and TOF-SIMS.

### 3.3. Analysis of Internal Spatial Structure

**Figure 6:**
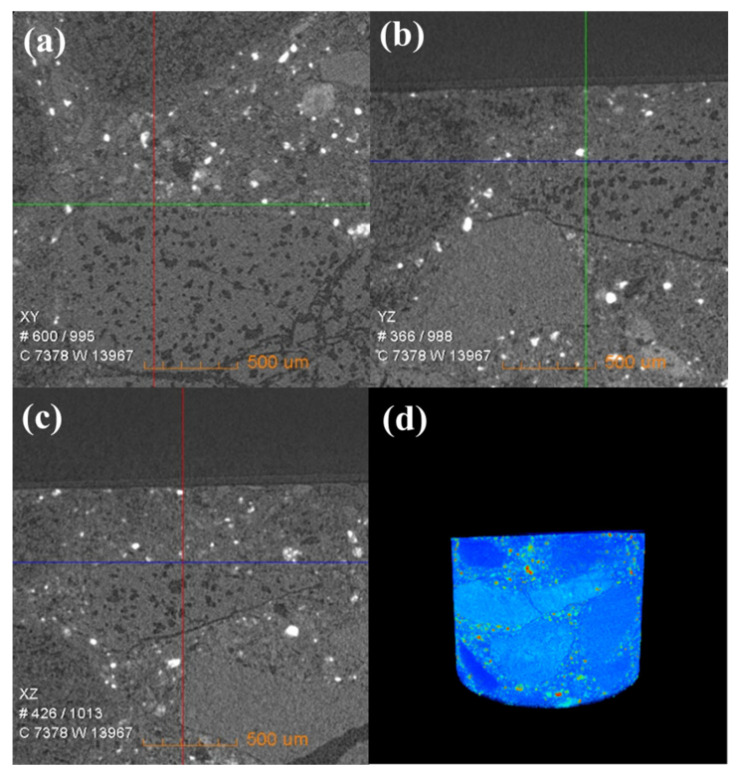
X-ray CT of a new saggar sample. (**a**), (**b**), and (**c**) Internal X-ray CT images of a new saggar sample scanned from the XY, YZ, and XZ directions, respectively. (**d**) Three-dimensional image in which bright and dark colors represent high and low density, respectively.

**Figure 7:**
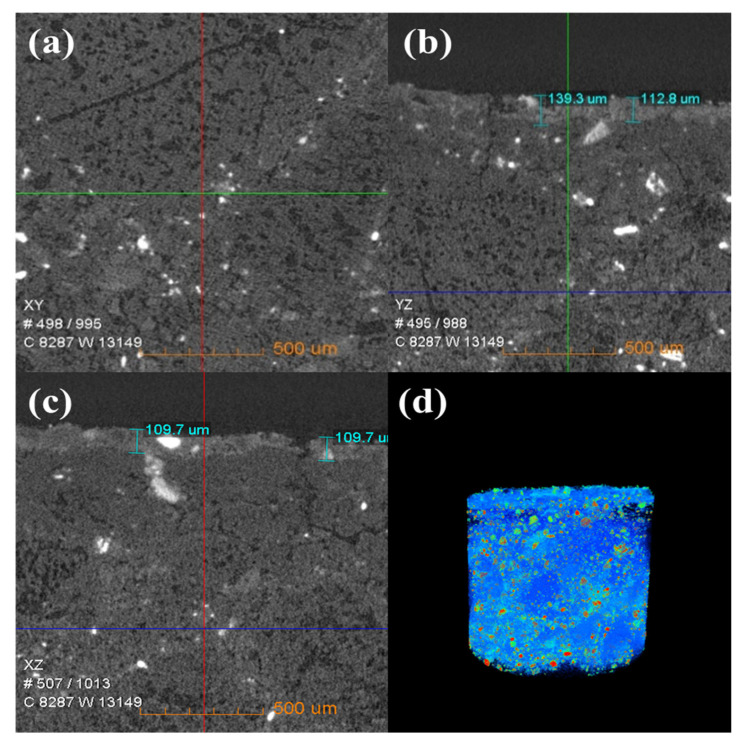
X-ray CT of a waste saggar sample. (**a**), (**b**), and (**c**) Internal X-ray CT figures of a waste saggar sample scanned from the XY, YZ, and XZ directions, respectively. (**d**) Three-dimensional image in which bright and dark colors represent high and low density, respectively.

Figure 6 shows the change in the internal density of new saggar and waste saggar characterized by X-ray CT analysis to further clarify the changes in the internal structure of the saggar before and after sintering. The bulk densities of cordierite, mullite, and corundum were 2.51 g/cm^3^, 3.156 g/cm^3^, and 3.5 g/cm^3^, respectively. Figure 6 shows that according to the XRD analysis results of new saggar, mullite and corundum aggregates were distributed in the interior of the phase as support, and fine alumina and cordierite were distributed between the aggregates as a binder phase. We also observed fine voids at the edge of auxiliary materials. In the cylinder section, except for the difference in aggregate density, the distribution of other dispersions in the ceramic was relatively uniform. Figure 7 shows the internal X-ray CT structure of the spent saggar obtained after sintering the LiCoO_2_ cathode material. A density-difference layer occurs on the contact surface between the saggar ceramic surface and the cathode material after the reaction. In the YZ and XZ surfaces, the thickness of the erosion reaction layer is approximately 110–140 μm, and many cracks can be found in the internal aggregate at the interface. This phenomenon is mainly due to the inconsistent expansion coefficient of lithium carbonate with mullite, cordierite, corundum, and other phases before and after reaction at high temperature. As a result, the thermal expansion coefficients of LiAlO_2_, LiAlSiO_4_, and Li_4_SiO_4_ were relatively large, leading to surface expansion and a large number of microcracks. The densities of LiAlO_2_, LiAlSiO_4_, and Li_4_SiO_4_ are 2.615 g/cm^3^ [19], 2.67 g/cm^3^ [20], and 2.35 g/cm^3^ [21], respectively, which are lower than those of mullite and corundum. Three-dimensional simulation images revealed that an erosion layer formed at the interface because of LiCoO_2_ and cobalt aluminate. The density of the part between the erosion and bulk layer was reduced due to the deep reaction of lithium, resulting in the formation of a low-density permeable reaction layer. Volume change in different areas caused by density change is one of the key reasons for saggar peeling or cracking.

### 3.4. Speculation on Reaction Mechanism and Verification

**Figure 8:**
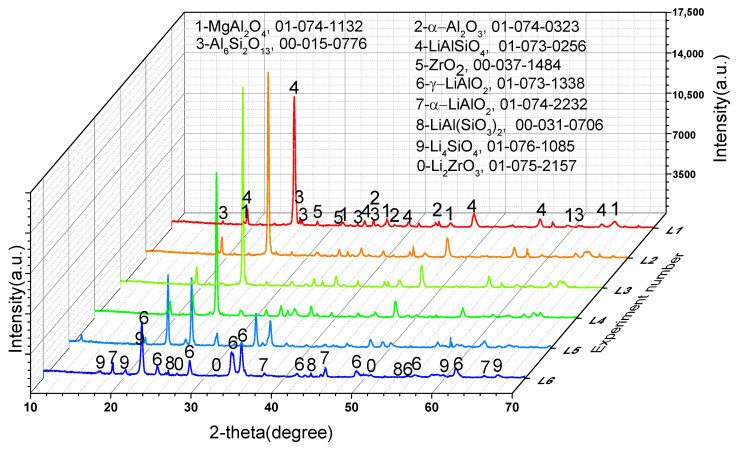
XRD spectra of the corrosion reaction of saggar powder with different contents of lithium carbonate.

To further confirm the phase transformation mechanism of the reaction process, we designed experiments on the reaction of Li_2_CO_3_ or Co_3_O_4_ with the crushed saggar powder to investigate the erosion mechanism. Figure 8 shows that when the consumption of lithium carbonate was low (experiment L1; Li_2_CO_3_: saggar powder mass ratio, 1:1), the reaction products were mainly LiAlSiO_4_, and the remaining phases were mainly unreacted mullite, cordierite, and corundum. We also found small amounts of Li_4_SiO_4_, ZrO_2_, and magnesium aluminum spinel in the product. With an increase in Li_2_CO_3_ consumption, the product’s content of lithium aluminosilicate gradually decreased, whereas that of γ-LiAlO_2_ and Li_4_SiO_4_ gradually increased. When lithium carbonate consumption was higher than the mass of saggar powder (experiment L6; Li_2_CO_3_: saggar powder mass ratio, 6:1), the reaction products were mainly γ-LiAlO_2_ and Li_4_SiO_4_, with small amounts of α-LiAlO_2_, lithium aluminum silicate, and lithium zirconate. With an increase in the quantity and length of reactions between the new saggar and raw materials, the amount of Li_2_CO_3_ on the saggar surface became highly excessive, with the main reaction products being lithium aluminate and lithium silicate.

**Figure 9:**
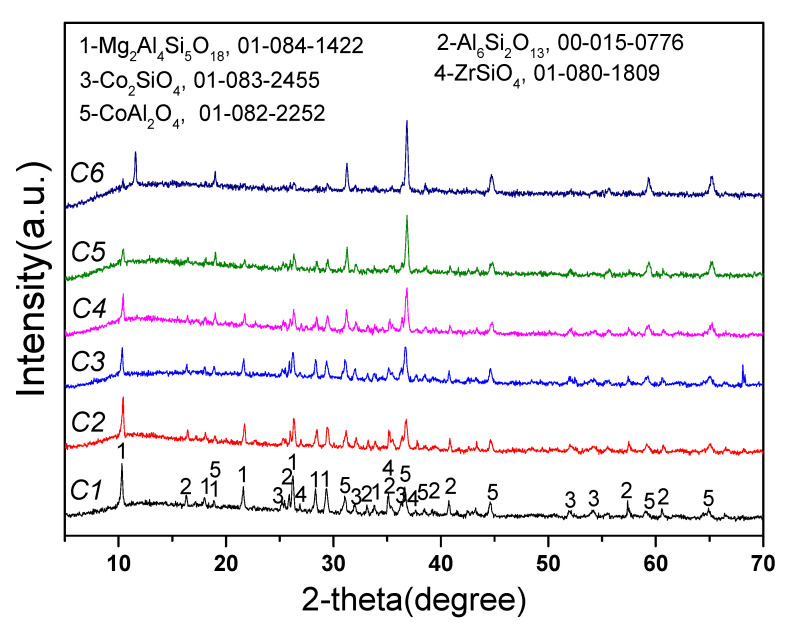
XRD spectra of the corrosion reaction of saggar powder with different contents of cobalt tetroxide.

Figure 9 shows that the main product phases from the reaction between Co_3_O_4_ and new saggar powder at 1000 °C were cobalt aluminate spinel and cobalt silicate. With the increase in Co_3_O_4_ dosage, the XRD spectra peak intensity of mullite, cordierite, and other main phases in the new saggar gradually decreased, whereas the peak intensity of the XRD spectra of cobalt aluminate spinel gradually increased. With the increase in Co_3_O_4_ consumption, the reaction products were mainly cobalt aluminate spinel and cobalt silicate. Some phases were not found in the waste saggar phase, mainly due to the slow penetration reaction of cobalt oxides and the small amount of phase generated on the contact surface.

**Figure 10:**
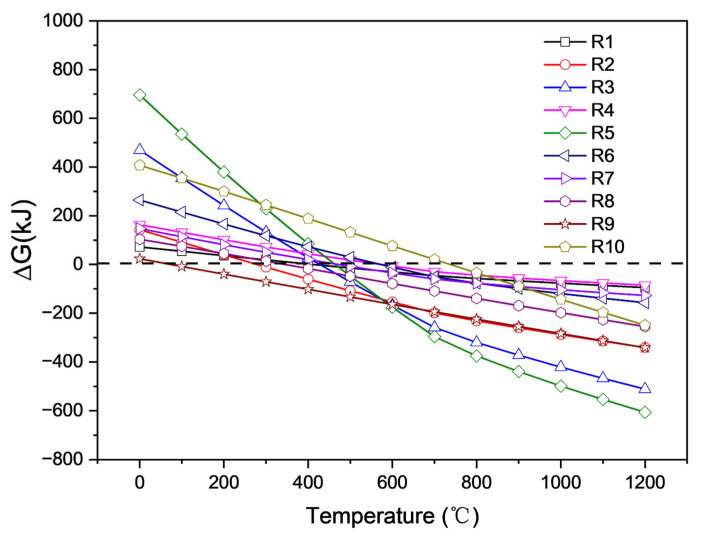
ΔG of different possible reactions of lithium carbonate and cobalt tetroxide with main components of ceramics at different temperatures obtained by HSC chemistry 6.0 calculation.

Figure 10 shows that at high temperatures, lithium carbonate appears to be a strong alkaline carbonate that can react with all components in saggar. All reactions may occur within the temperature range for the high-temperature synthesis of LiCoO_2_ cathode material. Among them, the reaction between Li_2_CO_3_ and cordierite phase was the easiest to occur and had the lowest activation energy at temperatures above 700 °C. In the case of sufficient Li_2_CO_3_, mullite, cordierite, and corundum particles can react with Li_2_CO_3_ to form lithium aluminate and lithium silicate. Reactions R1, R3, and R4 show that in the case of sufficient Li_2_CO_3_, most mullite, cordierite, and corundum particles can react with lithium carbonate to generate lithium aluminate and lithium silicate. The R2 chemical reaction formula shows that when the amount of lithium carbonate is small, the reaction of lithium carbonate with mullite forms a LiAlSiO_4_ phase. Reaction R4 indicates that lithium aluminosilicate further reacts with lithium carbonate to produce lithium aluminosilicate and lithium silicate. The ΔG of the different reactions shows that reaction R5 between cordierite and Li_2_CO_3_ is the most likely to occur, producing magnesium aluminum spinel, lithium silicate, and carbon dioxide. Reactions R6 and R7 show that when the content of lithium carbonate in the reactants is low, the solid products of ZrSiO_4_ and Li_2_CO_3_ are zirconia and lithium silicate. When the content of lithium carbonate in the reactants is high, the solid products of zirconium silicate and lithium carbonate are lithium zirconate and lithium silicate. The lithium aluminate products obtained by a high-temperature solid-state reaction exhibited two different phases, namely γ-LiAlO_2_ and α-LiAlO_2_. When the temperature is greater than 700 °C, α-LiAlO_2_ is transformed into γ-LiAlO_2_ [22]. Owing to the multiple cycles of high- and low-temperature conversion in the synthesis of the LiCoO_2_ cathode, the transformation reaction rate between γ-LiAlO_2_ and α-LiAlO_2_ was relatively slow, meaning different phases might coexist. When the synthesis temperature was above 1000 °C, γ-LiAlO_2_ occurred in the main phase of the lithium aluminate product. These results are consistent with our XRD analysis.

Reaction equations R8, R9, and R10 show the reaction behaviors of corundum, mullite, and cordierite, respectively, during the high-temperature reaction of cobalt oxide and the saggar body. The product phases are cobalt aluminum spinel and cobalt silicate, and our results are consistent with XRD analysis. Owing to the high molecular weight of cobalt and the low permeability of cobalt oxide in the saggar, the products generated by the reaction are mainly concentrated at the interface between the ceramic saggar materials and the cathode material. Therefore, the amounts of cobalt aluminum spinel and cobalt silicate are relatively low, and the corresponding peak intensity in the XRD spectrum of the waste saggar is relatively low.

The following reaction equations are the possible solid-state reactions at high temperature:

R1: Li_2_CO_3_ + Al_2_O_3_(C) = 2LiAlO_2_ + CO_2_(g)↑
R2: 3Li_2_CO_3_ + Al_6_Si_2_O_13_ = 2LiAlSiO_4_ + 3CO_2_(g)↑ + 4LiAlO_2_
R3: 7Li_2_CO_3_ + Al_6_Si_2_O_13_ = 2Li_4_SiO_4_ + 6LiAlO_2_ + 7CO_2_(g)↑
R4: LiAlSiO_4_ + 2Li_2_CO_3_ = LiAlO_2_ + Li_4_SiO_4_ + 2CO_2_(g)↑
R5: 10Li_2_CO_3_ + Mg_2_Al_4_Si_5_O_18_ = 5Li_4_SiO_4_ + 2MgAl_2_O_4_ + 10CO_2_(g)↑
R6: 3Li_2_CO_3_ + ZrSiO_4_ = Li_2_ZrO_3_ + 3CO_2_(g)↑ + Li_4_SiO_4_
R7: 2Li_2_CO_3_ + ZrSiO_4_ = ZrO_2_ + 2CO_2_(g)↑ + Li_4_SiO_4_
R8: 2Co_3_O_4_ + 6Al_2_O_3_(C) = 6CoAl_2_O_4_ + O_2_(g)↑
R9: 2Co_3_O_4_ + 1.2Al_6_Si_2_O_13_ = 1.2 CoAl_2_O_4_ + 2.4Co_2_SiO_4_ + O_2_(g)↑
R10: 3.333Co_3_O_4_ + Mg_2_Al_4_Si_5_O_18_ = 5Co_2_SiO_4_+ 1.666O_2_(g)↑ + 2MgAl_2_O_4_

The main reaction in the preparation of LiCoO_2_ cathode material is the corrosion of Li_2_CO_3_ on the ceramic body. With an increase in the number of saggar cycles, the amount of local lithium carbonate becomes excessive, resulting in the formation of lithium aluminosilicate, which reacts with lithium carbonate at high temperatures and, in turn, generates lithium silicate and lithium aluminate. The expansion coefficients of lithium silicate and lithium aluminate are higher than those of mullite and cordierite, leading to expansion between the erosion and bulk layers and resulting in cracking and peeling. These phenomena further result in saggar waste. On the one hand, the reaction between lithium carbonate and the saggar surface leads to the expansion of different phases, and the volume expansion leads to cracks between the reaction and bulk layers. On the other hand, the CO_2_ and O_2_ generated by the reaction further aggravate the expansion and cracking of the internal cracks of the material and the saggar interface [7,8,9]. Various factors are responsible for the waste of mullite–cordierite ceramic saggar. Subsequent preparation and improvement of new saggars must focus on solving the issues of lithium penetration and volume change in interfacial products. For the disposal and utilization of waste saggars, we must also consider the distribution of lithium to improve the overall waste-utilization efficiency. To improve the service life of saggar, the use of corrosion-resistant coatings should be increased to reduce the corrosion of lithium carbonate on the ceramic body.

## 4. Conclusions

In this study, we collected new and waste mullite–cordierite ceramic saggars and studied them by XRD and SEM-EDS. The surface erosion of saggars is the result of the combined reactions of lithium carbonate and cobalt oxide with mullite–cordierite ceramic material; we attributed the internal erosion of saggars mainly to lithium carbonate infiltration. We divided the interface contact area of waste saggar and the LiCoO_2_ cathode into four layers, namely the attached layer, hardened layer, permeability layer, and matrix layer. Our main findings are as follows:

(1) LiAlO_2_ and Li_4_SiO_4_ are the main phases after the reaction of sufficient lithium carbonate with aluminum compound. LiAlSiO_4_ and aluminum silicon lithium compounds are intermediate reaction products when lithium carbonate reactants are insufficient;
(2) TOF-SIMS analysis proves that the maximum erosion penetration of lithium can reach 2 mm. Cobalt erosion only occurs on the saggar’s surface, forming cobalt silicate and cobalt aluminum spinel;
(3) X-ray CT analysis shows that the internal reaction at the interface of lithium before and after the reaction leads to changes in the product’s density before and after the reaction, and the further volume change leads to saggar rupture. The CO_2_ and O_2_ generated by the reaction further aggravate the internal cracks of the material–saggar interface;
(4) The subsequent preparation of new, improved saggars must focus on solving issues regarding lithium penetration of interfacial products, which results in volume changes. Finally, for waste saggar disposal and utilization, lithium distribution should be considered to improve the overall utilization.

## Figures and Tables

**Table 1:** Experimental values and raw material consumption (g).

Sample	L1	L2	L3	L4	L5	L6	C1	C2	C3	C4	C5	C6
Saggar Powder	5	5	5	5	5	5	5	5	5	5	5	5
Li_2_CO_3_	5	10	15	20	25	30	/	/	/	/	/	/
Co_3_O_4_	/	/	/	/	/	/	5	10	15	20	25	30

## Data Availability

The data used to support the findings of this study are available upon request from the corresponding author.

## References

[1] Zhao Y., Yuan X., Jiang L., Wen J., Wang H., Guan R., Zhang J., Zeng G. (2020). Regeneration and reutilization of cathode materials from spent lithium-ion batteries. Chem. Eng. J..

[2] Min X., Sun B., Chen S., Fang M., Wu X., Liu Y.G., Abdelkader A., Huang Z., Liu T., Xi K. (2019). A textile-based SnO2 ultra-flexible electrode for lithium-ion batteries. Energy Storage Mater..

[3] Min X., Xiao J., Fang M., Wang W., Zhao Y., Liu Y., Abdelkader A.M., Xi K., Kumar R.V., Huang Z. (2021). Potassium-ion batteries: Outlook on present and future technologies. Energy Environ. Sci..

[4] Liao Z., Zhang S., Li K., Zhang G., Habetler T.G. (2019). A survey of methods for monitoring and detecting thermal runaway of lithium-ion batteries. J. Power Sources.

[5] Porthault H., Cras F.L., Franger S. (2010). Synthesis of LiCoO2 thin films by sol/gel process. J. Power Sources.

[6] Ning Chen B.L., Gui W., Xu S., Wang J., Dai J. (2018). Research on temperature field change trend of the sintering process for lithium-ion battery cathode materials. IFAC-PapersOnLine.

[7] Duan X., Zheng H., Chen Y., Qian F., Liu G., Wang X., Si Y. (2020). Study on the corrosion resistance of cordierite-mullite and SiC refractories to Li-ion ternary cathode materials. Ceram. Int..

[8] Zhai P.T., Chen L.G., Yin Y.M., Li S.P., Ding D.F., Ye G.T. (2018). Interactions between mullite saggar refractories and Li-ion battery cathode materials during calcination. J. Eur. Ceram. Soc..

[9] Zhai P., Chen L., Hu S., Zhang X., Ding D., Li H., Li S., Zhu L., Ye G. (2019). Comparison of interactions of MgO-based refractories with Li-ion battery cathode materials during calcination. Int. J. Appl. Ceram. Tec..

[10] Huang H., Huang Z., Fang M., Liu Y., Jiang B. (2011). Erosion Resistance of Magnesia-alumina Spinel in Synthesis Process of Lithium Cobaltaoxide. Bull. Chin. Ceramic. Soc..

[11] Ding D., Ye G., Chen L. (2019). Superior corrosion resistance KAlSi2O6-containing materials for calcining Li-ion battery cathode materials. Corros. Sci..

[12] Ding D., Chen L., Liao G., Zheng L., Gao S., Ye G. (2019). Preparation of andalusite-corundum-KAlSi2O6 material for the calcination of Li-ion battery cathode materials. J. Alloys Compd..

[13] Kakroudi M.G., Vafa N.P., Asl M.S., Shokouhimehr M. (2020). Effects of SiC content on thermal shock behavior and elastic modulus of cordierite–mullite composites. Ceram. Int..

[14] Wilson P.J., Blackburn S., Greenwood R.W., Prajapti B., Smalley K. (2011). The role of zircon particle size distribution, surface area and contamination on the properties of silica–zircon ceramic materials. J. Eur. Ceram. Soc..

[15] Kazemi A., Faghihi-Sani M.A., Nayyeri M.J., Mohammadi M., Hajfathalian M. (2014). Effect of zircon content on chemical and mechanical behavior of silica-based ceramic cores. Ceram. Int..

[16] Cornette P., Zanna S., Seyeux A., Costa D., Marcus P. (2020). The native oxide film on a model aluminium-copper alloy studied by XPS and ToF-SIMS. Corros. Sci..

[17] Hu P., Hou X., Zhang J., Li S., Wu H., Damø A.J., Li H., Wu Q., Xi X. (2018). Distribution and occurrence of lithium in high-alumina-coal fly ash. Int. J. Coal Geol..

[18] Sjövall P., Bake K.D., Pomerantz A.E., Lu X., Mitra-Kirtley S., Mullins O.C. (2021). Analysis of kerogens and model compounds by time-of-flight secondary ion mass spectrometry (TOF-SIMS). Fuel.

[19] Tsai S.C., Chen H.C., Huang J.C., Chang C.M., Chou M.M.C. (2016). Size and orientation effect on the mechanical properties of LiAlO2 single crystal. Mater. Sci. Eng. A.

[20] Qi J., Ba J., Li H., Lin J., Zheng X., Cao J., Feng J. (2020). β-LiAlSiO4 reinforced Cu composite interlayer for brazing C/C composites and Nb. Vacuum.

[21] Pejchal J., Babin V., Beitlerova A., Kurosawa S., Yokota Y., Yoshikawa A., Nikl M. (2017). Improvement of the growth of Li4SiO4 single crystals for neutron detection and their scintillation and luminescence properties. J. Cryst. Growth.

[22] Isupov V.P., Bulina N.V., Borodulina I.A. (2018). Effect of the reaction medium on the mechanochemical synthesis of LiAlO2. Inorg. Mater..

